# Participation and performance trends of East-African runners in Swiss half-marathons and marathons held between 2000 and 2010

**DOI:** 10.1186/2052-1847-5-24

**Published:** 2013-12-01

**Authors:** Marco Cribari, Christoph A Rüst, Thomas Rosemann, Vincent Onywera, Romuald Lepers, Beat Knechtle

**Affiliations:** 1 Institute of General Practice and for Health Services Research, University of Zurich, Zurich, Switzerland; 2 Department of Recreation Management and Exercise Science, Kenyatta University, Nairobi, Kenya; 3 INSERM U1093, Faculty of Sport Sciences, University of Burgundy, Dijon, France; 4 Facharzt FMH für Allgemeinmedizin, Gesundheitszentrum St. Gallen Vadianstrasse 26, St. Gallen 9001, Switzerland

**Keywords:** Running, East African runners, Nationality, Aging, Gender, Marathon

## Abstract

**Background:**

This study examined the changes in participation, performance and age of East African runners competing in half-marathons and marathons held in Switzerland between 2000 and 2010.

**Methods:**

Race times, sex, age and origin of East African versus Non-African finishers of half-marathon and marathon finishers were analyzed.

**Results:**

Across time, the number of Kenyan and Ethiopian finishers remained stable (*P* > 0.05) while the number of Non-African finishers increased for both women and men in both half-marathons and marathons (*P* < 0.05). In half-marathons, the top ten African women (71 ± 1.4 min) and top three (62.3 ± 0.6 min) and top ten (62.8 ± 0.4 min) African men were faster than their Non-African counterparts (*P* < 0.05). In marathons, however, there was no difference in race times between the top three African men (130.0 ± 0.0 min) and women (151.7 ± 2.5 min) compared to Non-African men (129.0 ± 1.0 min) and women (150.7 ± 1.2 min) (*P* > 0.05). In half-marathons and marathons was no difference in age between the best Non-African and the best African runners (*P* > 0.05).

**Conclusions:**

During the last decade in Switzerland, the participation of Kenyan and Ethiopian runners in half- and full- marathons remained stable. In marathons there was no difference in age and performance between the top African and the top Non-African runners. Regarding half-marathons, the top African runners were faster but not younger than the top Non-African runners. Future insight should be gained by comparing the present results with participation, performance and age trends for East African runners competing in marathons held in larger countries.

## Background

Over the last years, participation and performance trends in long-distance running such as half- marathon and marathon running have been extensively analyzed [1-12]. It has been demonstrated that age [1-6], sex [2,4-7], training [2,4,8], anthropometry [2,4,8] and nationality [9] were crucial determining factors for long-distance running performance.

For instance, it has been shown for the ‘New York City Marathon’ that participation increased in the last decades [1,2] especially for women [6] and master athletes [1]. The participation of master athletes – defined as athletes older than 35 years [10] – increased in half-marathons [2,11], marathons [1,2,6,11,12] and ultra-marathons [13]. Leyk et al. [2,4] suggested that health promoting behaviour and the ability of finishing a marathon with only a short running history might be a potential explanation for this phenomenon. More than 25% of master athletes aged between 50 and 69 years started running no more than five years before their first participation in a marathon [4]. They further reported that a high number of running kilometres and high running frequencies were needed to successfully finish a marathon compared to their younger counterparts [4]. Nevertheless, running performance is related to age-dependent physiological changes [3,10,14-19]. Recent studies showed that best marathon times in general population will be achieved before the age of forty years [1,3,6].

The question of a relationship between performance and origin in marathon runners has also been raised [8,9,20-23]. In addition, the top list of the International Association of Athletics Federations (IAAF) in half-marathon and marathon runners [24] in 2011, especially for men, indicated that all the top 20 performances in marathons and half-marathons were achieved by East African runners originating from Kenya, Ethiopia and Eritrea. The dominance of these East African athletes in long-distance running is a well-known phenomenon [9,25]. Specific advantageous factors such as favourable genetic endowment [9] and a better running economy [8,21,22] have been suggested for the success of East African runners. The anecdotal finding of travelling a long way to school each day was postulated as another important factor for the great success of East African runners. Both Onywera et al. [26] and Scott et al. [25] reported that most of the national and international elite runners from Kenya and Ethiopia run every day more than five kilometres to school. They further showed an association between the distance travelled to school and the performance in long-distance running [25,26].

Beside these factors, also unique sociocultural and psychological conditions were suggested as an important reason of the success of East African runners [9,22,26]. Kenyans have an advantageous motivation to become a competitive athlete [26,27]. Poverty and unemployment are major problems in most African countries [26,27]. Therefore, running might be the only chance for a successful life and to raise money in order to help their families and parents [26,27]. Supported by western countries, running became more professional in Kenya and Ethiopia [8,26,27]. So in the last years the possibility of both earning money and becoming a successful runner increased [8,26,27]. In western countries with a high gross domestic product (GDP) such as Switzerland and a large general income [28], we assume a greater sponsorship at several larger races comparing to other countries with a lower GDP. Therefore, earning money with promotional fees and prize money could be an important motivation for East African elite runners to compete in Swiss long-distance running events.

To the best of our knowledge, the changes in participation and performance trends of East African runners have not been analyzed in all half-marathon and marathon races held within an entire country. Therefore, the aims of this study were to analyze (*i*) the participation trends of East African *versus* Non-African runners in all Swiss half-marathons and marathons held between 2000 and 2010, and (*ii*) the trends in performance and the age of peak performance of African and Non-African athletes for both women and men during the studied period. Regarding the general increase in participation in long-distance running events and the increasing influence of western countries in Kenyan and Eritrean running culture, we hypothesized an increase in the participation of East African runners. Based on previous reports of East-African dominance in long-distance running, we also hypothesized better performances for top African runners compared to top Non-African runners. According to this and based on age-related peak performances in long-distance running events, we further assumed that top African runners would be younger than top Non-African runners in both half-marathons and marathons over the last decade.

## Methods

All runners who ever finished a half-marathon and a marathon in Switzerland between 2000 and 2010 were analyzed with the focus on participation, sex, nationality and age. The data set for this study was obtained from http://www.datasport.ch and the race directors. The study was approved by the Institutional Review Board of St. Gallen, Switzerland, with waiver of the requirement for informed consent given that the study involved the analysis of publicly available data.

During this period, data from 313,173 athletes were available with 226,754 finishers in half-marathons and 86,419 finishers in marathons from a total of 90 marathons and 145 half-marathons. Out of these two groups, eight subgroups (*i.e.* four subgroups for each distance) were generated and sorted by nationality (*i.e.* African or Non-African) and sex (*i.e.* men or women). Africans, *i.e.* Ethiopians and Kenyans, were analyzed more specifically. In order to find trends in the annual number of male and female finishers per race distance, the number of finishers was determined and analyzed for African and Non-African athletes separately. Since Ethiopia and Kenya were the two African nations providing the highest number of African finishers, the participation trends for athletes originating from these two countries were in addition analyzed separately. For Non-African finishers, no detailed information about their nationality was extracted. Therefore, data from 226,518 Non-African athletes (*i.e.* 162,288 men and 64,230 women) and 236 African athletes (*i.e.* 169 men and 67 women) were available in half-marathons. In marathons, data from 86,182 Non-African athletes (*i.e.* 71,200 men and 14,982 women) and 237 African athletes (*i.e.* 183 men and 54 women) could be analysed (Table 1). In order to compare African with Non-African athletes, the overall top (*i.e.* fastest race time), the top three (*i.e.* three fastest race times) and the top ten (*i.e.* ten fastest race times) male and female athletes were determined for African and Non-African athletes in both half-marathons and marathons during the studied period. The athletes were then analyzed and compared regarding their race performance and age.

**Table 1:** History of half-marathons and marathons held in Switzerland between 2000 and 2010 with the number of African and Non-African finishers

**Half-marathon**	**African men**	**African women**	**Total Africans**	**Non-African men**	**Non-African women**	**Total Non-Africans**	**Total runners**
Greifenseelauf	69	32	101	57,847	24,978	82,825	82,926
Lausanne Marathon	14	2	16	23,497	8,946	32,443	32,459
Hallwilerseelauf	40	17	57	22,461	8,489	30,950	31,007
Lucerne Marathon	1	2	3	10,880	5,665	16,545	16,548
Maratona Ticino	3		3	10,038	2,588	12,626	12,629
Genève Marathon for UNICEF	30	8	38	8,636	3,561	12,197	12,235
Winterthur Marathon	2		2	6,284	2,322	8,606	8,608
Basler Marathontage/Run to the Beat				3,880	1,439	5,319	5,319
Dreiländerlauf Basel				3,822	983	4,805	4,805
Bieler Lauftage	2	1	3	2,930	1,030	3,960	3,963
Brienzerseelauf				1,935	943	2,878	2,878
Rhylauf	3	2	5	2,043	535	2,578	2,583
Run und Walk Event Rothenburg				1,343	532	1,875	1,875
Halbmarathon SM 2000 Schwyz	2	1	3	1,278	296	1,574	1,577
Virgin Runners Halbmarathon				1,118	332	1,450	1,450
Sempachersee Halbmarathon				765	312	1,077	1,077
Basel City				728	344	1,072	1,072
Emmentaler Halbmarathon				617	193	810	810
Pfäffikerseelauf				634	142	776	776
Neujahrsmarathon				386	208	594	594
Halbmarathon Schweizermeisterschaft 2002 Brittnau	3	2	5	396	93	489	494
Wiedlisbacher Halbmarathon				203	80	283	283
Cuorsa dil Rein Disentis				164	62	226	226
Semi marathon Côte de l'orbe				131	72	203	203
Int. Halbmarathon Full-Reuenthal				119	35	154	154
Halbmarathon Gürbetal				106	38	144	144
Sri Chinmoy Self Transcendence Marathon Winterthur Halbmarathon				47	12	59	59
**Total Half-marathon**	**169**	**67**	**236**	**162,288**	**64,230**	**226,518**	**226,754**
**Marathon**							
Zürich Marathon	96	24	120	31,277	6,799	38,076	38,196
Lausanne Marathon	59	23	82	13,017	2,606	15,623	15,705
Lucerne Marathon	2	1	3	7,063	1,713	8,776	8,779
Genève Marathon for UNICEF	17	3	20	4,319	758	5,077	5,097
Winterthur Marathon		2	2	4,297	798	5,095	5,097
Maratona Ticino	2		2	2,607	399	3,006	3,008
Basel City	4	1	5	2,288	572	2,860	2,865
Basler Marathontage/Run to the Beat	1		1	1,809	336	2,145	2,146
Frauenfeld				1,649	342	1,991	1,991
Bieler Lauftage				1,529	374	1,903	1,903
Rund um den Bielersee				752	164	916	916
Neujahrsmarathon Schlieren	2		2	590	120	710	712
Sri Chinmoy Self Transcendence Marathon Winterthur				3	1	4	4
**Total Marathon**	**183**	**54**	**237**	**71,200**	**14,982**	**86,182**	**86,419**

### Statistical analysis

In order to increase the reliability of our data analysis, each set of data was tested for normal distribution as well as homogeneity of variances in advance. Normal distribution was tested using a D’Agostino and Pearson omnibus normality test, homogeneity of variances using a Levene’s test in case of two groups and with a Bartlett’s test in case of more than two groups. In order to find significant changes in a variable across years, single linear regression analysis was used. To find significant differences between two groups, a Student’s *t*-test was used. Statistical analyses were performed using IBM SPSS Statistics (Version 19, IBM SPSS, Chicago, IL, USA) and GraphPad Prism (Version 5, GraphPad Software, La Jolla, CA, USA). Significance was accepted at *P* < 0.05 (two-sided for *t*-tests). Data in the text are given as mean ± standard deviation (SD).

## Results

### Participation trends

**Figure 1:**
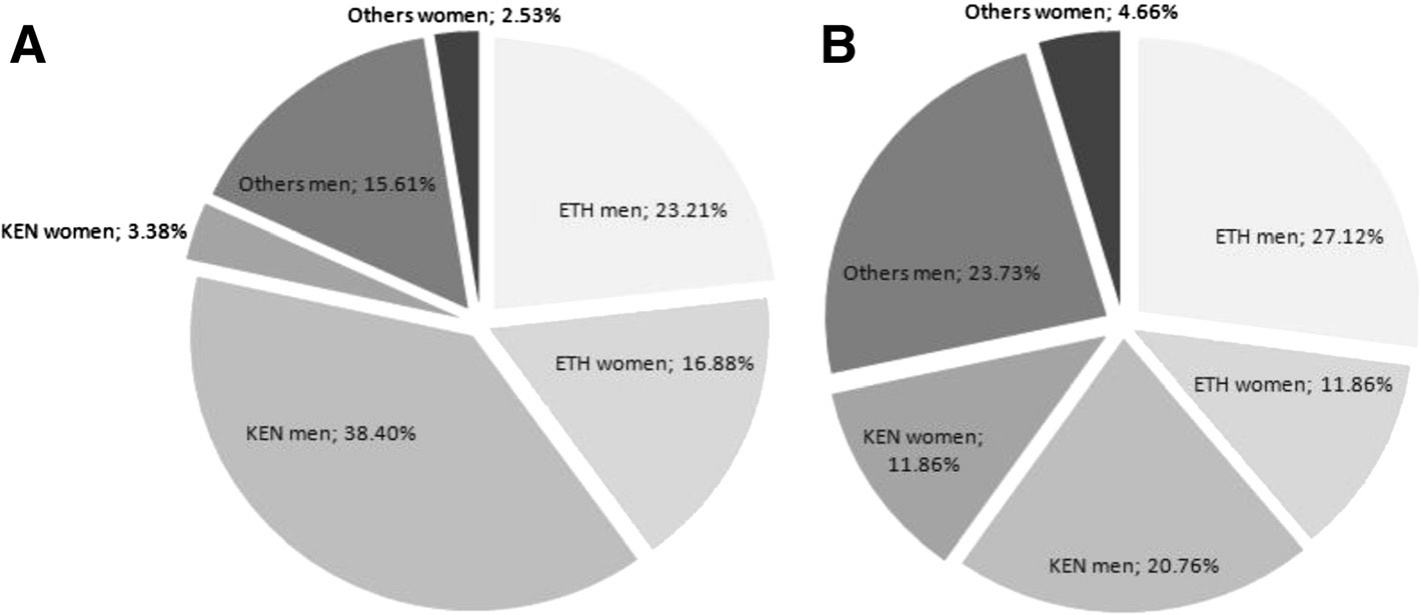
**Percent of Ethiopian and Kenyan men and women in overall African runners for marathons (Panel A) and half-marathons (Panel B).** Ken = Kenya, ETH = Ethiopia.

In marathons, 0.2% of all finishers were African men and 0.1% African women during the 2000–2010 period (Table 2). In African finishers, 23.3% (overall finishers: 0.1%) were Ethiopian men, 38.4% (0.1%) Kenyan men, 16.9% (0.1%) Ethiopian women and 3.4% (0.01%) were Kenyan women (Figure 1A). In half-marathons, 0.1% of all finishers were African men and <0.1% were African women (Table 1). In African finishers, 27.1% (overall finishers: <0.1%) were Ethiopian men, 20.8% (<0.1%) were Kenyan men, 11.9% (<0.1%) were Ethiopian women and 11.9% (<0.1%) were Kenyan women (Figure 1B).

**Table 2:** Absolute and relative number of finishers in marathons and half-marathons in Switzerland sorted by nationality and gender during the 2000–2010 period

	**Ethnicity**	**Gender**	**Number of finishers**	**Percent of finishers**
Marathon			86,419	100.00%
	Non-African	Men	71,200	82.39%
	Non-African	Women	14,982	17.34%
	African	Men	183	0.21%
	Ethiopian	Wen	55	0.06%
	Kenyan	Men	91	0.11%
	African	Women	54	0.06%
	Ethiopian	Women	40	0.05%
	Kenyan	Women	8	0.01%
Half-marathon			226,754	100.00%
	Non-African	Men	162,288	71.57%
	Non-African	Women	64,230	28.33%
	African	Men	169	0.07%
	Ethiopian	Men	64	0.03%
	Kenyan	Men	49	0.02%
	African	Women	67	0.03%
	Ethiopian	Women	28	0.01%
	Kenyan	Women	28	0.01%

**Figure 2:**
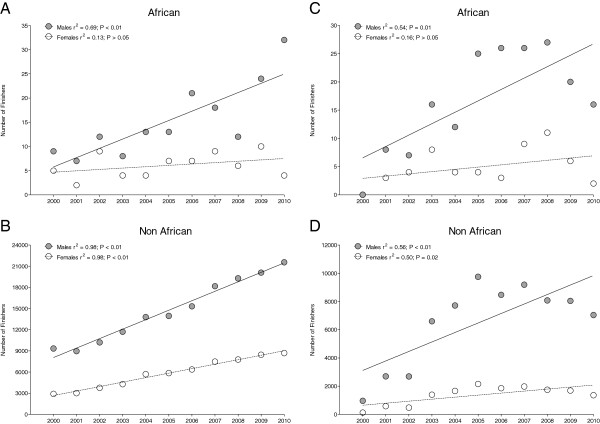
Change in the number of male and female African (Panels A&C) and Non-African (Panels B&D) finishers in half-marathon (Panels A&B) and marathon (Panels C&D), respectively, during the 2000–2010 period.

In half-marathons, the number of Non-African male finishers (14,753 ± 4,492) was higher than the number of Non-African female finishers (5,839 ± 2,122 finishers) (*P* < 0.05). For African men, the annual number of half-marathon finishers increased (*P* < 0.01) (Figure 2A). The number of Non-African male and female finishers increased over the last decade in half-marathons by 131.1% for men and by 199.6% for women (*P* < 0.01) (Figure 2B). In marathons, the number of Non-African male finishers (6,473 ± 2,966) was higher than the number of Non-African female finishers (1,362 ± 670.7) (*P* < 0.05). Only African men increased their annual number of marathon finishers (*P* = 0.01) (Figure 2C). Non-African men (*P* < 0.01) and women (*P* = 0.02) increased their annual number of marathon finishers by 160.9% for men and by 134.4% for women over the last decade (Figure 2D). In half-marathons, there was no difference between the number of African male (15.4 ± 7.7) and African female (6.1 ± 2.5) finishers (*P* > 0.05). Also for marathons, there was no difference between the number of African male (16.6 ± 9.1) and African female (4.9 ± 3.3) finishers (*P* > 0.05).

### Participation trends of Ethiopian and Kenyan athletes

**Figure 3:**
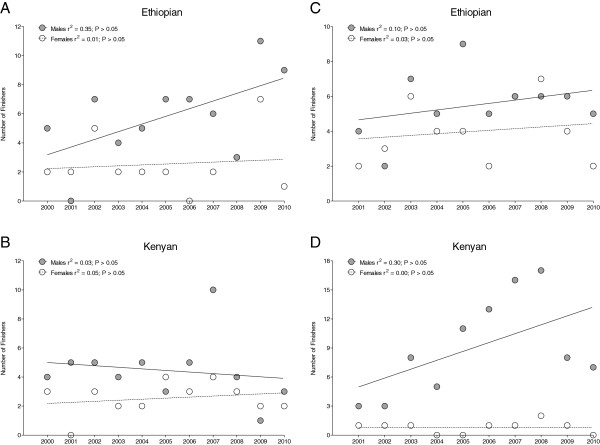
Change in the number of male and female Ethiopian (A&C) and Kenyan (B&D) finishers in half-marathon (A&B) and marathon (C&D), respectively, during the 2000–2010 period.

In half-marathons, the annual number of male Ethiopian finishers (5.8 ± 3) was higher (*P* < 0.05) than the annual number of female Ethiopian (2.5 ± 1.9) finishers (Figure 3A). In Kenyan athletes, there was no difference (*P* > 0.05) between the annual number of male (4.5 ± 2.2) and female (2.5 ± 1.1) finishers (Figure 3B). In Ethiopian as well as in Kenyan finishers, neither men nor women showed a significant change in the number of annual finishers across years (*P* > 0.05). In marathons, the mean number of male Ethiopians (5.5 ±1.8) was not different (*P* > 0.05) from the number of female Ethiopians (4 ± 1.8) (Figure 3C). In Kenyans, the number of male finishers (9.1 ± 5) was higher (*P* < 0.05) than the number of female finishers (0.8 ± 0.6) (Figure 3D). In both Ethiopians and Kenyans, neither men nor women showed a significant change in the number of finishers across the years (*P* > 0.05).

### Participation trends for specific races held in Switzerland

**Figure 4:**
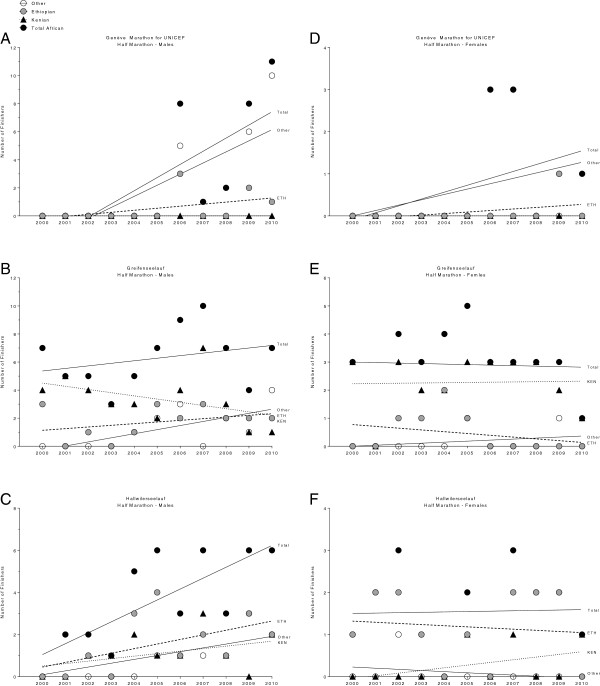
Change in participation for female and male East African runners in specific half-marathons in Switzerland for men in Genève Marathon for UNICEF (Panel A), Greifenseelauf (Panel B) and Hallwilerseelauf (Panel C) and for women in Genève Marathon for UNICEF (Panel D), Greifenseelauf (Panel E) and Hallwilerseelauf (Panel F).

**Figure 5:**
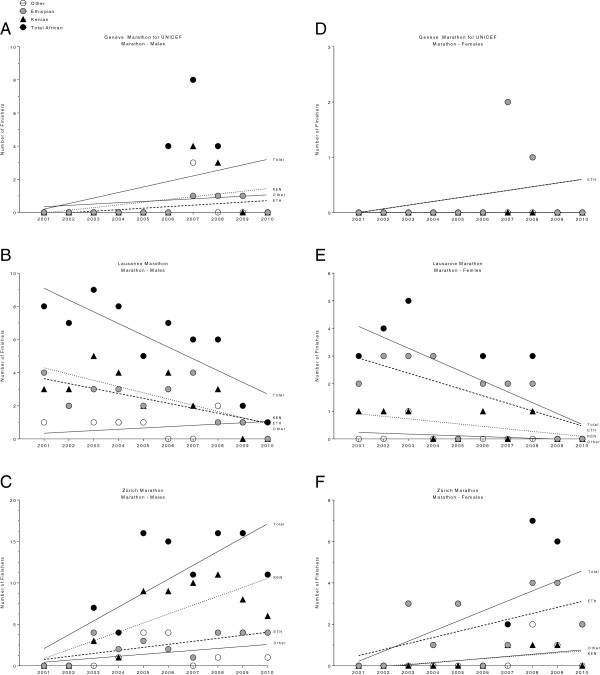
Change in participation in specific marathons in Switzerland for female and male East African runners for men in Genève Marathon for UNICEF (Panel A), Lausanne Marathon (Panel B) and Zürich Marathon (Panel C) and for women in Genève Marathon for UNICEF (Panel D), Lausanne Marathon (Panel E) and Zürich Marathon (Panel F).

The change in participation for East African runners in specific races held in Switzerland is presented in Figure 4 for half-marathons and in Figure 5 for marathons. For men, a significant decrease in both Ethiopian and Kenyan marathoners in ‘Lausanne Marathon’ was found (Figure 5B) whereas for women, only the number of Ethiopian athletes decreased (Figure 5E). In contrast, the number of both male Ethiopian and male Kenyan runners increased in the same period in ‘Zurich Marathon’ (Figure 5C).

### Comparison of African and Non-African runners’ performance

**Figure 6:**
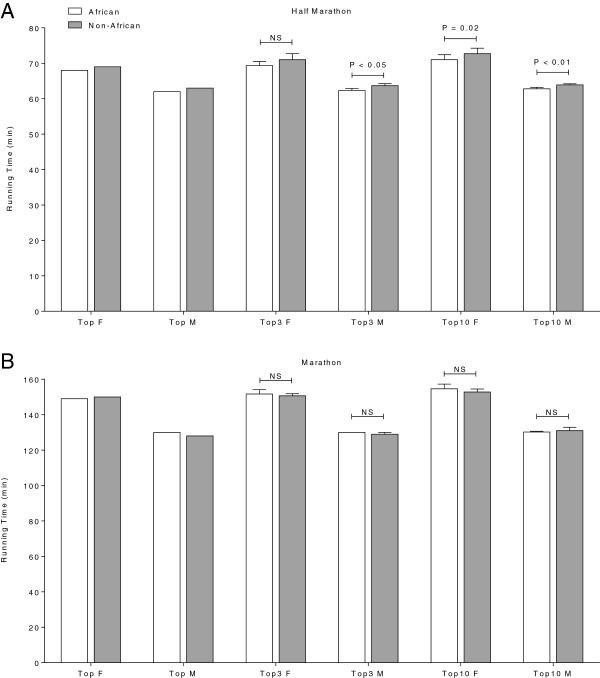
**Running time of the overall top, the overall top three and the overall top ten African and Non-African women and men in half-marathon (Panel A) and marathon (Panel B).** P-values indicate whether the difference between African and Non-African athletes was significant or not (‘NS’ = not significant).

In half-marathons, the overall top three race times of African women (69.3 ± 1.2 min) were not faster than those of Non-African women (71 ± 1.7 min). In contrast, the top ten race times in African women (71 ± 1.4 min) were faster than those of Non-African women (72.7 ± 1.6 min). African men were faster than Non-African men regarding the top three (*P* = 0.04) and the top ten (*P* < 0.01) race times (Figure 6A).

The average race times for African men were 130 ± 0 min for the top three and 130.2 ± 0.4 min for the top ten. Compared to top three (129.0 ± 1.0 min) and top ten (131.0 ± 1.9 min) race times in Non-African men there was no difference in performance (*P* > 0.05). The same was found for women (*P* > 0.05). The African top three women finished in 151.6 ± 2.5 min compared to Non-African top three women 150.6 ± 1.2 min. The mean race time for top ten African women was 154.6 ± 2.7 min compared to 152.8 ± 1.7 min for top ten Non-African women (Figure 6B). Comparing the race times of all African and Non-African finishers in half-marathons and marathons, African women (126 ± 52 min) were faster than Non-African women (145 ± 54 min) (*P* < 0.01). The average race time of all female finishers was 145 ± 54 min. Comparing African men (113 ± 49 min) and Non-African men (143 ± 62 min) there was also no difference in performance (*P* < 0.01). The mean race time of all male finishers was 143 ± 62 min. Comparing all finishers, men and women, African athletes (116 ± 50 min) were faster than Non-African athletes (144 ± 60 min) (*P* < 0.01). The mean finishing time of all finishers was 144 ± 60 min (Table 3).

**Table 3:** Race time and number of Africans and Non-Africans finishers in marathons and half-marathons between 2000 and 2010 in Switzerland

	**Non-African**		**African**		**Overall**	
	**Race time (min)**	**Number of finishers**	**Race time (min)**	**Number of finishers**	**Race time (min)**	**Number of finishers**
Women	145 ± 54	79,212	126 ± 52	121	145 ± 54	79,333
Men	143 ± 62	233,488	113 ± 49	352	143 ± 62	233,840
Total	144 ± 60	312,700	116 ± 50	473	144 ± 60	313,173

Results are presented as mean ± SD.

### Comparison of African and top Non-African runners age

**Figure 7:**
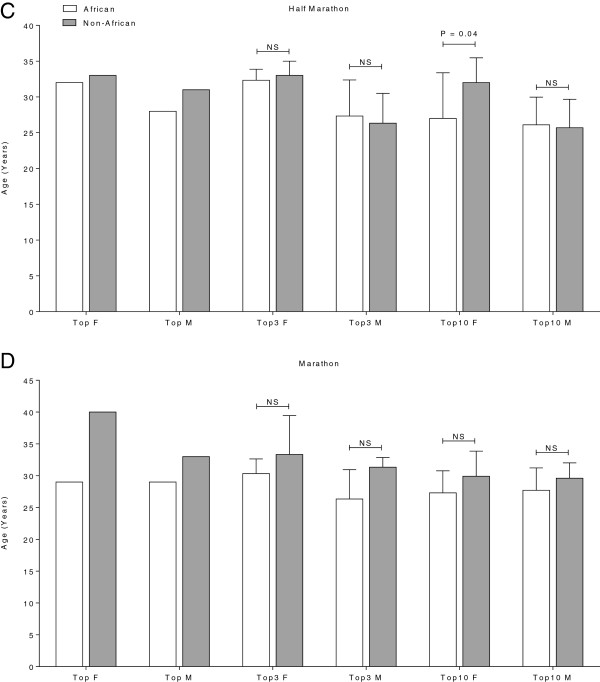
**Age of the overall top, the overall top three and the overall top ten African and Non-African women and men in half-marathon (Panel C) and marathon (Panel D).** P-values indicate whether the difference between African and Non-African athletes was significant or not (‘NS’ = not significant).

In half-marathons, the top three African women (32.3 ± 1.5 years) and men (27.3 ± 5 years) were not younger than the top three Non-African women (33 ± 2 years; *P* = 0.67) and men (26.3 ± 4.2 years; *P* = 0.80). Also in men’s top ten there was no difference in age between African (26.1 ± 3.9 years) and Non-African (25.7 ± 4 years) athletes (*P* = 0.82). Comparing the top ten finishers for African (27 ± 6.4 years) and Non-African (32 ± 3.5 years) women, African women were younger (*P* = 0.04; Figure 7A) than Non-African women. In marathons, comparing the top three and top ten finishers, there was no difference in age between African and Non-African men, and between African and Non-African women (Figure 7B).

## Discussion

The first aim of the present study was to analyze participation trends of East African runners in Swiss half-marathons and marathons held between 2000 and 2010. The second aim was to compare performance and age of African runners and Non-African runners. The main findings were (*i*) a stable annual number of Kenyan and Ethiopian finishers in both half- and full-marathons while the number of Non-African finishers increased for both men and women, (*ii*) a higher number of male African finishers compared to female African finishers, (*iii*) faster race times for African athletes in half-marathons for both men and women, (*iv*) similar race times for Africans and Non-Africans for both sexes for marathons, and (*v*) no difference in the age of peak performance for top African and top Non-African runners for both sexes in half-marathons and marathons.

### Participations of East African runners remained stable in the last decade

In contrast to our hypothesis, the number of Kenyan and Ethiopian finishers remained stable for both men and women in both half-marathons and marathons. The increase of African men in half-marathon and marathon was due to other African countries, which were not analysed in details. In contrast, the numbers of male and female finishers in Non-Africans increased in both marathons and half-marathons. This finding correlates with results presented by Jokl et al. [6] reporting an increased participation in general over the last 30 years in a large city marathon such as the ‘New York City Marathon’.

Regarding the studied running events, African mainly competed at larger marathon and half-marathon events (*e.g.* Greifenseelauf, Lausanne Marathon, Hallwilerseelauf, Genève Marathon for UNICEF and Zürich Marathon). In all other races, there were five or fewer Africans competing during this period. We assume for smaller events lower budgets and these events were less prestigious. Interestingly, the ‘Lucerne Marathon’, the ‘Maratona Ticino’ and the ‘Winterthur Marathon’ were all among the seven largest running events for half-marathons and marathons. However, fewer than four Africans were participating annually per race between 2000 and 2010. In personal conversation with the race directors from the ‘Lucerne Marathon’ and the ‘Winterthur Marathon’ we learned that prize money was less than 200 Swiss francs (*i.e.* about 220 US dollars) and no promotional fees were offered either for African or for Non-African athletes. They further reported a decline of the prize money over years leading to less attraction for elite African athletes. Therefore, they races become more attractive for regional marathoners and half-marathoners. So in common beliefs the opportunity to win the race increased in absence of the elite East African athletes. Other personal communications with the race directors of the ‘Greifenseelauf’, the ‘Hallwilerseelauf’, the ‘Zurich Marathon’ and the ‘Lausanne Marathon’ (*i.e.* races with a high number of African participants) revealed that special prize money and promotional fee (especially for board and lodge) for elite African athletes were offered. They specially invited elite athletes in general to make their competition more interesting. Perhaps from the ‘Maratona Ticino’ and the other races no data for prize money were available and beside the ‘Winterthur Marathon’ only from the ‘Lausanne Marathon’ an amount of the prize money was given [29].

According to poverty and unemployment in East African countries, Onywera [27] and Onywera *et al.*[26] reported that the motivation to become a competitive athlete was mainly based on economic reasons for Kenyan elite athletes especially for 39% of all national athletes and 33% for international athletes. The international success of the East African running pioneers such as Mike Boit, Kipchoge Keino and others implicated a ‘westernation’ of the culture, especially in Kenya [27]. Therefore, running became more professional and more popular in Kenya. Affected by the great international success of the East African running pioneers, young athletes know about the possibility of becoming a successful international athlete. Motivation of competitive running therefore increased and young East-African athletes became support by managers of Western countries. So the East African athletes came closer to the Western civilisation and according to this theory of closeness the chance to make money with competitive running increased. Based on this sociocultural background and according to our different findings in participation of East African athletes in Swiss long-distance running events and personal conversation with the race directors the question about a correlation between prize money and participation went up. In addition, the respondent race directors from running events with a large number of Africans reported a stable budget for invited elite athletes. Therefore, the possibility to make money with competitive running in Switzerland is limited and the annual number of Kenyan and Ethiopian participants remains stable. For further studies it would be interesting to analyse potential correlations between prize money and participation of African elite athletes in larger countries (*e.g.* USA).

### Kenyan and Ethiopian participation

During the studied period there were more African men than African women competing. The same can be reported for Non-African athletes. Participation differences between men and women are common findings in sports events. In 1988 at the Olympic Games in Seoul (KOR) only 26.1% of all participants were women [30]. Compared to the XXIX Olympic Games in Beijing (CHN) in 2008 women increased their percentage of participants up to 42.4% [30]. So, especially in earlier years there were restrictions on female athletes. In Kenya, women were banned from international competitions until the 1970s [27]. Regarding the percentage of female participants at the Olympic Games in Seoul, 5.5% of the Kenyan team were women. They increased their participation also up to 39.1% in the Olympic Games in Beijing (2008) [31]. Therefore, the female running culture has not such a long time history as that of men. The Kenyan men were well established in long-distance running since years. But today, a female running culture is growing in Kenya and it is also a great honour for female athletes to represent their country in international competitions [27]. Participation of Kenyan woman increased with a delay of several years compared to Kenyan men. Also in Ethiopia, there was a similar development. Comparing Olympic Games in 1980 (Ethiopia took not part in the Olympic Games in 1988 and 1984) and 2008, Ethiopian women increased their participation from 4.9% to 48.1% [31]. These reports may explain the difference between female and male participation trends of Kenyan athletes in marathons and Ethiopian athletes in half-marathons.

More interesting seems the fact of the difference in participation in male Ethiopian and Kenyan athletes in both half-marathons and marathons. Compared to their Ethiopian counterparts, more Kenyan men were participating in marathons and fewer in half-marathons. A possible explanation for this might be differences in anthropometry between Kenyan and Ethiopian runners. Ethiopians are more mesomorphic in somatotype, which includes more muscle mass especially expressed in a high thigh circumference [32]. Kenyans in contrast are more ectomorphic with slender legs [32]. Zillmann *et al.*[33] showed differences in anthropometric characteristics between recreational marathoners and recreational half-marathoners competing in Switzerland. They reported for half-marathoners a thicker thigh circumference compared to marathoners [33]. To the best of our knowledge anthropometric characteristics of Ethiopian runners have not been analysed yet. Based on reported differences in anthropometry between Ethiopians and Kenyans [32] we can only speculate that Ethiopian runners were more predestined for running half-marathons than marathons. Therefore, we had a higher number of Ethiopian participants in half-marathons than in marathons. Also in the IAAF top list from 2011 in marathons and half-marathons there was a significant difference between the number of Ethiopian and Kenyan runners for the top 20 race times [24]. In the top 20 marathoners there were 20 Kenyans [24]. In half-marathons there were 13 Kenyans and 9 Ethiopians ranked [24]. Future studies need to analyse anthropometric differences between Ethiopian and Kenyan long-distance runners.

In the two largest marathons held in Switzerland, the ‘Zurich Marathon’ and the ‘Lausanne Marathon’, a reverse trend was found regarding the participation of male East African marathoners. While the participation decreased in the ‘Lausanne Marathon’, it increased in the ‘Zurich Marathon’. Most probably the prize money was higher in ‘Zurich Marathon’ compared to ‘Lausanne Marathon’ during this period.

### Elite African marathoners were not faster than elite Non-African marathoners

There was no difference in race times between elite African finishers and elite Non-African finishers in marathons. This finding does not support the hypothesis of a better running performance of elite African runners compared to Non-African runners and was not in line with the anecdotal well-known African dominance in the literature regarding long-distance running [8,9,20-23]. Comparing maximum oxygen uptake (VO_2_max) between elite Kenyan and elite Non-African runners, disparate findings were reported [8,21-23]. Therefore, VO_2_max as an important predictor variable of performance was critically discussed and in general there is no difference between Kenyan and Non-African runners [8]. Also, no differences were found in proportion of muscle fibers and muscle oxidative enzymes [8,21]. Running economy, defined as the steady-state of submaximal oxygen uptake at a given running velocity, was further critically discussed as an important factor of performance [8]. Saltin *et al.*[34] reported a better running economy for Kenyans while Bosch *et al.*[35] reported no difference in the oxygen uptake at given performance levels. In summary, no significant physiological differences between elite African runners and elite Non-African runners could have been found yet and this goes in line with our unique results in marathons.

Nevertheless, regarding the IAAF top list of marathons and half-marathons and corresponding to common beliefs, East African runners had better running performances than Non-African runners [24]. Why do marathon events in Switzerland differ to marathons held in other countries? A plausible explanation might be the thesis that not the top of the top East African athletes were competing in Swiss long-distance running events because they were less prestigious and there was less prize money to win. For example in the Lausanne marathon there were about 2,150 U.S. Dollar (*i.e.* 2,000 Swiss francs) for winning the marathon [29]. Comparing to the ‘New York City Marathon’ as the most prestigious marathon event worldwide the winner got about 100’000 U.S. Dollars [36]. To confirm this thesis about ‘second class’ elite East African runners in Swiss marathons further studies should analyze East African performance trends in larger countries. Especially performance findings of East African athletes from the World Marathon Majors (*i.e.* a collective of the worlds’ largest marathon events) should be compared to our data of smaller country with smaller races.

### Top African runners were faster than top Non-African runners in half-marathons

Interestingly, elite African athletes were faster than elite Non-African athletes in half-marathons. This finding reflects the general opinion, anecdotal findings and international top lists of the IAAF [24] and goes not in line with our results found for marathons. Based on scientific literature we cannot explain this fact with physiological differences between Africans and Non-Africans. An explanation might be different sociocultural backgrounds and differences in running experience. A recent study compared a variety of predictor variables for performance between half-marathons and marathons [33]. The authors reported that marathoners had a larger number of completed long-distance competitions in their running history than half-marathoners. So in Switzerland, marathoners start their running career with half-marathons to gain experience [33]. Relatively to the world records 2011 in half-marathons and marathons [37] Non-African half-marathoners differ to Non-African marathoners in race times. The top Non-African race time in men’s half-marathons was 7.3% away of the world record. In marathons, however, there was only a gap of 3.4%. In Swiss long-distance events Non-African marathoners were better than half-marathoners in the worldwide ranking. More newcomers and recreational runners were competing in Swiss half-marathons might be a possible explanation for this. Non-African recreational runners were motivated for running by health benefits [2,4]. It is possible to run a favorable performance beside a workaday life because not as many training kilometers and training units are needed for half-marathons compared to marathons [33] which is essential in Switzerland. Therefore, with a lot of newcomers in Non-Africans the possible top race time have not been reached yet. In contrary, top African runners were professional runners with a large running history since childhood and they were also driven by economical reasons to compete in Swiss half-marathons [25-27]. So, they run better performances compared to Non-African athletes in half-marathons.

### Elite African runners were not younger than elite Non-African runners

In contrast to our second hypothesis, elite African finishers were not younger than elite Non-African finishers neither in half-marathons nor in marathons. First, the majority of African runners in Switzerland between 2000 and 2010 were elite East African runners (76.7%) from Ethiopia (39.5%) and Kenya (37.21%). Performance is limited by age-related physiological changes. The maximal oxygen uptake (VO_2_max), as one of the most important factors, declines with age [3,10,14,16,18]. In addition to VO_2_max, the maximum heart rate (HR_max_), the lactate threshold and the running economy change with age. HR_max_ significantly decreases with age [10,15,16], the lactate threshold, as the exercise intensity (%VO_2_max), increases significantly with age [10,17,19]. Top performances in long-distance running events will be achieved at younger ages (<40 years) [1,3,6] for both Africans and Non-Africans. Therefore, we can explain the same age of top performances comparing African and Non-African athletes in marathons and half-marathons. Especially in half-marathons, there was no age difference but a significant difference in performance between Africans and Non-Africans. This could be explained by an age-dependent loss in performance as well. The correlation between age and performance is relatively and not absolutely. So, within each group peak performance will be achieved at the same age regardless of a difference in performance between these two groups. The difference in performance would be explained by the fact that Non-African athletes have not reached their possible peak performance yet and not by age-differences.

### Strength, weakness, limitations and implications for future research

A strength of the present study is the high number of runners analyzed in marathons and half-marathons from one country for an entire decade. To date, a demographical analysis about participation in marathons and half-marathons in such a way is unique. However, this study is limited in that determining factors for performance such as training, anthropometry, physiology, nutrition and motivation were not included. A limitation is also that data about prize money only was given in personal conversations with the race directors. Future studies should investigate participation, age and performance trends in marathoners and half-marathoners in larger countries such as the United States of America in order to strengthen demographic research. In addition, psychological and sociocultural backgrounds of the motivation of East African runners in relation to participation trends in half marathons and marathons should be analyzed in-depth.

## Conclusion

Participation in East African runners remained stable over the last decade in Swiss half-marathons and marathons while the number of Non-African men and women increased. There was no difference in race times of the top three African and the top three Non-African marathoners. The dominance of East-African runners in marathons documented in literature remains anecdotal for marathons held in Switzerland between 2000 and 2010. Future studies need to analyze participation, age and performance trends of East-African runners in larger countries such as the United States of America to become a better insight of the East-African runners phenomena in long-distance running.

## Competing interests

The authors declare that they have no competing interests.

## Authors’ contributions

All authors designed the study. The manuscript was written by all authors. All authors read and approved the final manuscript.

## Pre-publication history

The pre-publication history for this paper can be accessed here:

http://www.biomedcentral.com/2052-1847/5/24/prepub
